# Perinatal mortality among infants born during health user-fees (Cash & Carry) and the national health insurance scheme (NHIS) eras in Ghana: a cross-sectional study

**DOI:** 10.1186/s12884-016-1179-2

**Published:** 2016-12-08

**Authors:** Abdallah Ibrahim, Ernest T. Maya, Ernestina Donkor, Irene A. Agyepong, Richard M. Adanu

**Affiliations:** 1 School of Public Health, University of Ghana, Accra, Ghana; 2 School of Nursing, University of Ghana, Accra, Ghana; 3 Ghana Health Service, Dodowa Health Research Center, Dodowa, Ghana

**Keywords:** Perinatal mortality, Northern Region, NHIS, Tamale Teaching Hospital, Cash and Carry

## Abstract

**Background:**

This research determined the rates of perinatal mortality among infants delivered under Ghana’s national health insurance scheme (NHIS) compared to infants delivered under the previous “Cash and Carry” system in Northern Region, especially as the country takes stock of its progress toward meeting the Millennium Development Goals (MDG) 4 and 5.

**Methods:**

The labor and maternity wards delivery records of infants delivered before and after the implementation of the NHIS in Northern Region were examined. Records of available daily deliveries during the two health systems were extracted. Fisher’s exact tests of non-random association were used to examine the bivariate association between categorical independent variables and perinatal mortality.

**Results:**

On average, 8% of infants delivered during the health user-fee (Cash & Carry) died compared to about 4% infant deaths during the NHIS delivery fee exemption period in Northern Region, Ghana. There were no remarkable difference in the rate of infant deaths among mothers in almost all age categories in both the Cash and Carry and the NHIS periods except in mothers age 35 years and older. Infants born to multiparous mothers were significantly more likely to die than those born to first time mothers. There were more twin deaths during the Cash and Carry system (*p* = 0.001) compared to the NHIS system. Deliveries by caesarean section increased from an average of 14% in the “Cash and Carry” era to an average of 20% in the NHIS era.

**Conclusion:**

The overall rate of perinatal mortality declined by half (50%) in infants born during the NHIS era compared to the Cash and Carry era. However, caesarean deliveries increased during the NHIS era. These findings suggest that pregnant women in the Northern Region of Ghana were able to access the opportunity to utilize the NHIS for antenatal visits and possibly utilized skilled care at delivery at no cost or very minimal cost to them, which therefore improved Ghana’s progress towards meeting the MDG 4, (reducing under-five deaths by two-thirds).

## Background

Every year, an estimated four million perinatal deaths occurs worldwide [1]. Perinatal mortality, as used in this study, consists of stillbirths and deaths occurring within the first week of life [1]. Almost all (98%) of these perinatal deaths occur in low -middle income countries (LMIC) including Sub-Saharan Africa [1, 2]. As a LMIC in sub-Saharan Africa, Ghana has had its fair share of these mortalities, especially in parts of the country where there are challenges with access to supervised delivery.

The United Nation’s Millennium Development Goal four (MDG4) is a reduction in the deaths of infants and children under-5 years by two-thirds by the end of 2015 with reference to levels in 1990 [3]. Achieving MDG 4 in Ghana has been one of the factors that led to the country’s implementation of a health system that ensured unfettered access to antenatal care services, which is crucial to reducing the rate of infant deaths. Recent data indicates that infant and neonatal mortality rates for Ghana are estimated to be 53 and 32 per 1000 live births respectively with regional variations [4]. The Northern Region has infant and neonatal mortality rates of 66 and 39 per 1000 live births respectively which are all higher than the national average [4].

Antenatal care provides the opportunity to detect and address complications early in pregnancy to avoid some of these adverse outcomes. It also affords pregnant women the opportunity to receive valuable counselling and other interventions such as malaria prophylaxis, tetanus toxoid, iron and folate supplementation that increases the chances of their infant’s survival.

Not surprising therefore, lack of antenatal care has been associated with adverse pregnancy outcomes including prematurity, low birth weight, fetal and neonatal deaths, even when delivery takes place in hospital [5]. In Ghana, antenatal care is known to reduce the risk of neonatal death by approximately 2% [6].

In the 1990s and early 2000s, Ghana had a health user fee system known as “Cash and Carry” that demanded the payment of fees before services were rendered, creating barriers to health care for many who needed it the most [7]. In 2003, Ghana introduced a National Health Insurance Scheme (NHIS) that exempted fees for prenatal and delivery care for expectant mothers in the poorest four of the country’s ten regions [8]. In the years since the pilot, the NHIS was extended to the rest of the country for open enrolment. By removing the financial barriers to antenatal care, the NHIS delivery exemption program offers the opportunity to examine birth outcomes under the two systems, particularly the perinatal mortality of facility-based deliveries in the Northern Region where issues of poverty, access to skilled care, societal beliefs and perception of perinatal mortality are somewhat linked. Although enrolment in the NHIS program had dropped in some districts in Ghana, enrolment among the indigent in the Northern Region increased in excess of 100% by the end of 2008 [9, 10]. The increased in NHIS enrolment among the indigent in Northern Region ensured that women got access to antenatal care and skilled care at delivery at health facilities, which is known to influence perinatal deaths in sub-Saharan Africa [6].

In many African communities, perinatal deaths are so common that some people in these communities generally tend not to consider them as a major public health problem. In these communities, including those in Northern Region, there is a general perception of not fully recognizing the birth of an infant as complete until the newborn survives the first week of life when the child’s naming ceremony (commonly called *out-dooring*) takes place to formally and traditionally *out-door* the infant to the entire community [1, 11]. Therefore, many infant deaths in places such as Northern Region go unaccounted for because of such cultural perceptions that do not encourage reporting of such deaths, especially since the perception of infants not reaching full week after birth makes them unqualified as being counted as a complete birth [1].

The current study was designed to compare trends in perinatal mortality among birth of infants during Ghana’s national health insurance delivery fee exemption program and full-fee paying (Cash & Carry) systems. Specifically, the study determined the rates of perinatal mortality in infants born under the NHIS exemption program compared to infants born under the former Cash and Carry system at the Tamale Teaching Hospital (TTH), the only teaching hospital in the entire Northern Ghana. It has been noted that in Northern Ghana, only 13% of neonatal deaths occur in hospitals [12].

The Northern Region, which is considered one of the most deprived areas in Ghana, is known to have higher incidence of poverty compared to the rest of the country. During the hospital fee-paying period in the 1990s, poverty rates in the Northern Region had increased from 63% in 1991/92 to 69% in 1998/99, compared to the Greater Accra Region in the south that decreased from 26 to 5% in the same period [13].

Even in recent years after the introduction of the NHIS, Northern Region is still noted as having the highest incidence of multi-dimensional poverty index (MPI—the amount of deprivations that poor households experience at a point in time) compared to the rest of the country [14]. Recent estimates indicate that between 2006 and 2010, the Northern Region in particular had higher poverty incidence rate of 80.9% compared to the other two regions in Northern Ghana, including Upper East Region (80.8%) and Upper West Region (77.6%), or the rest of the country [14]. Women in Northern Region tend to experience disproportionately high levels of poverty due to several factors including: bearing the majority of household economic burdens; engaging in trading and farming activities with limited access to capital; discriminatory land ownership customs; polygamous households; and high rates of illiteracy [15].

The high rates of poverty in Ghana discussed earlier, and the even higher rates in the northern part of the country where there is least availability of health services results in the worst public health outcomes in that part of the country, including infant deaths [16, 17].

## Methods

This cross-sectional study used birth records in Northern Region of Ghana before and after the introduction of the country’s NHIS program. The study data was extracted from the birth registry folders located at the labour and maternity wards of the Tamale Teaching Hospital (TTH). Tamale, which is the regional capital of the Northern Region, is the fourth largest metropolitan city in Ghana. It has a population of less than 400,000 (50% are females), and an annual population growth rate of about three percent [18, 19].

The sampling technique used to collect the birth records was a purposive sampling method, which was done to ensure that records of available daily birth of infants were represented in the total sample from the maternity ward’s delivery folders. The selection process was done by chronologically arranging the folders of birth records of infants born at the Tamale Teaching Hospital by year and by month of birth so that up to seven birth records would be extracted for each day. However, in year 2000, there were only 363 complete records available and for that matter they were all selected at the time of data collection. Sampling from the available daily birth of infants included mainly women of the Mole-Dagomba ethnic group who delivered their newborns at the hospital. The hospital generally serves people mainly from the entire northern part of the country but more specifically those in the metropolitan Tamale and the surrounding areas. The Northern Region is a predominantly Moslem population with a mixture of Christians and other traditional believers who are in the minority. Majority of the different ethnic groups in the region tend to have more socio-economic and cultural similarities than differences [20].

The total sample used for this study was 8312 birth records. It included 3957 birth records for infants delivered under the Cash and Carry system between 2000 and 2003 (comparison group) and 4355 records of infants delivered between 2008 and 2011 representing the NHIS period (intervention group). Although the minimum sample required for this study was 1890 birth records (945 NHIS births and 945 Cash and Carry births), based on the assumptions: α = 0.05 (two-sided); β = 0.20; and power = 0.80 and an estimated 25% reduction in perinatal mortality, the total sampled records extracted from the birth folders were used for the study.

The dependent or outcome variable of interest was ‘perinatal mortality’ of infants born at the Tamale teaching hospital. This outcome variable was coded as a dichotomous variable so that live birth (infant survived) and perinatal mortality (still-birth and infant death within the first week of life) yielded precise and desired outcomes in repeated measurements.

The primary independent variable of interest was the model of health financing available for maternal and newborn care in Ghana’s health system at the time of infant’s ’ delivery. This predictor variable (i.e. model of health financing) was categorized as either NHIS or “Cash and Carry” health system. The category of infant births during the NHIS period was used as a proxy for access to insurance or free maternal delivery and at least four recommended antenatal care visits “Cash and Carry” was a proxy for out-of-pocket payment (or delivery during health user-fee) and limited or no antenatal care.It had already been established in other studies that more than 90% of pregnant women who delivered their infants during the NHIS system received antenatal care services, with the majority of them receiving the World Health Organization’s recommended four visits prior to delivery of their infants [21, 22]. Covariates in this study were: maternal age, parity (number of prior deliveries); obstetric haemorrhage (blood loss after delivery measured in millilitres); infant’s sex; mode of delivery (vaginal or caesarean), nature of birth (singleton or twin); and fetal heart rate (measured in beats per minute or bpm). Unfortunately, some variables known to affect perinatal mortality, e.g., gestational age, were not available from the birth records. Some of the covariates such as blood loss and fetal heart rate were also missing some values. Other important variables not captured in the delivery folders at the hospital included socio-economic factors. These variables were therefore not available and were not included in the study.

Fisher’s exact tests were done to examine any non-random association between the categorical independent variables and perinatal mortality from the birth records. Fisher’s exact test was used for the study’s analysis because some of the cells had less than five variable units. Adjusted logistic regression models were also done to estimate the odds ratios for perinatal mortality in infants born during the cash and carry and NHIS periods. All the other variables in the model were included in the multiple regression analysis to estimate the adjusted odds ratios. These other variables, including maternal age, maternal parity, type and nature of birth, sex, post-partum hemorrhage (blood loss) and fetal heart beat were all included in the model because they are all known to be linked to adverse birth outcomes, including infant deaths, hence none was excluded [23–25]. The study’s analyses were done using Stata, version 11.2 (Stata Corp, College Station, TX).

## Results

A total sample of 8312 birth records was used for the study analysis. Of that, 3957 births (48%) were from the “Cash and Carry” system and 4355 (52%), NHIS. Table 1 displays the characteristics of all sampled births during both periods at the Tamale Teaching Hospital. The mean age of mothers in both the “Cash and Carry” and NHIS periods was 27 years and a standard deviation of about 6 years. The youngest mother was 14 years old and the oldest was 50. Mean parity was less than two prior births and the maximum parity was eleven.

**Table 1:** Characteristics of infants born at a tertiary hospital facility during the “Cash and Carry” and national health insurance scheme (NHIS) systems in Northern Region

Variables	Cash & Carry period				NHIS period			
	n(%)				n(%)			
	2000	2001	2002	2003	2008	2009	2010	2011
Annual births(N):	363	1223	1196	1175	1100	1109	1046	1100
Mother's age								
< 18	2(1)	19(2)	17(1)	14(1)	16(2)	10(1)	10(1)	6(1)
18–24	124(34)	401(33)	414(35)	374(32)	333(30)	344(31)	293(28)	321(29)
25–34	186(51)	628(51)	573(48)	616(52)	597(54)	578(52)	606(58)	626(57)
≥ 35	51(14)	175(14)	192(16)	171(15)	154(14)	177(16)	137(13)	147(13)
Parity								
None	122(34)	411(34)	448(37)	406(35)	388(35)	358(32)	347(33)	376(34)
One	67(18)	245(21)	222(19)	237(20)	222(20)	224(20)	221(21)	220(20)
Two	50(14)	177(15)	169(14)	178(15)	185(17)	192(17)	176(17)	192(17)
Three	31(9)	155(13)	137(12)	146(13)	134(12)	120(11)	120(11)	151(14)
Four-plus	54(15)	209(17)	219(18)	198(17)	170(15)	214(19)	165(16)	154(15)
Type of birth								
Caesarean	39(11)	148(12)	167(14)	209(18)	198(18)	199(18)	218(21)	250(23)
Vaginal	324(89)	1075(88)	1029(86)	966(82)	902(82)	910(82)	828(79)	850(77)
Nature of birth								
Singleton	335(92)	1117(91)	1084(91)	1079(92)	994(90)	1013(91)	964(92)	974(89)
Twin	28(8)	106(9)	112(9)	96(8)	106(10)	96(9)	82(8)	126(11)
Sex								
Male	204(56)	667(55)	625(52)	637(54)	575(52)	571(51)	561(54)	553(50)
Female	159(44)	556(45)	571(48)	538(46)	525(48)	538(49)	485(46)	547(50)
Blood loss after delivery								
< 200	281(77)	170(14)	137(11)	127(11)	209(20)	209(19)	312(20)	343(31)
200–250	13(4)	338(28)	574(48)	574(49)	304(28)	446(40)	299(29)	274(25)
≥ 250	3(1)	477(39)	257(21)	208(18)	65(6)	47(4)	258(25)	65(6)
Fetal heart rate								
< 130	8(2)	45(4)	124(10)	143(12)	111(10)	134(12)	77(7)	69(6)
130–140	215(59)	751(61)	711(59)	714(61)	709(64)	610(55)	707(68)	692(63)
≥ 140	25(7)	82(7)	95(8)	107(9)	51(5)	129(12)	43(4)	112(10)

Table 2 presents the Bivariate analysis of perinatal mortality among infants in the Cash and Carry and NHIS systems. Overall, about 8% of infants born during the Cash and Carry system died, compared to an average of 4% during the NHIS period in the Northern Region of Ghana. There were no remarkable differences observed in the rate of infant deaths among mothers in all age categories in both the Cash and Carry and the NHIS systems except in mothers aged 35 or more who lost more infants during the Cash and Carry period (*p* = 0.001) compared to mothers in the same age group under NHIS (*p* = 0.504). There were fewer twin deaths observed during the NHIS period compared to the Cash and Carry era. Mothers with three prior birth experiences during the Cash and Carry period significantly lost more infants in the perinatal period compared to similar mothers who gave birth during the NHIS period. On average, less than 14% of infants were born through Caesarean method during the Cash and Carry period compared to 20% Caesarean births during the NHIS period. Between 18 and 42% (*p* = 0.004) of infant deaths occurred under Caesarean deliveries during the NHIS compared to 14–35% (*p* = 0.001) infant deaths among Caesarean delivered infants during the Cash and Carry period.

**Table 2:** Bivariate analysis of perinatal mortality among infants delivered at a tertiary hospital facility during “Cash and Carry” and NHIS periods in Northern Region

Variables	Cash & Carry period					NHIS period				
	n(%)					n(%)				
	2000	2001	2002	2003	Fisher’s exact	2008	2009	2010	2011	Fisher’s exact
Annual mortalities	28(8)	105(9)	71(6)	112(9)		60(5)	44(4)	56(5)	33(3)	
Mother's age										
< 18	0(0)	6(6)	0(0)	2(2)	0.001	3(5)	0(0)	0(0)	0(0)	0.504
18–24	10(36)	28(26)	23(32)	29(26)		22(37)	10(23)	17(31)	10(30)	
25–34	14(50)	45(43)	29(41)	61(54)		22(37)	28(63)	31(55)	19(58)	
≥ 35	4(14)	26(25)	19(27)	20(18)		13(21)	6(14)	8(14)	4(12)	
Parity										
None	7(28)	34(33)	22(31)	36(34)	0.001	21(35)	6(13)	21(38)	11(34)	0.278
One	7(28)	11(11)	9(13)	14(13)		10(17)	11(25)	11(20)	3(9)	
Two	2(8)	8(8)	8(11)	14(13)		6(10)	11(25)	8(14)	6(18)	
Three	5(20)	20(20)	12(17)	22(20)		10(17)	4(9)	6(11)	5(15)	
Four-plus	4(16)	29(28)	20(28)	22(20)		13(21)	12(27)	9(16)	8(24)	
Type of birth										
Caesarean	4(14)	20(19)	22(31)	39(35)	0.001	20(33)	8(18)	15(27)	14(42)	0.004
Vaginal	24(86)	85(81)	49(69)	73(65)		40(67)	36(82)	41(73)	19(58)	
Nature of birth										
Singleton	24(86)	80(76)	57(80)	100(89)	0.001	56(93)	40(91)	52(93)	2(6)	0.376
Twin	4(14)	25(24)	14(20)	12(11)		4(7)	4(9)	4(7)	31(94)	
Sex										
Male	15(54)	53(51)	31(44)	66(59)	0.556	27(45)	24(55)	31(55)	13(39)	0.825
Female	13(46)	52(49)	40(56)	46(41)		33(55)	20(45)	25(45)	20(61)	
Post-partum hemorrhage(ml)										
< 200	20(91)	17(22)	7(15)	19(28)	0.013	8(31)	8(23)	16(46)	10(59)	0.247
200–250	1(4.5)	29(37)	17(38)	36(52)		14(54)	25(74)	11(31)	4(23)	
≥ 250	1(4.5)	32(41)	21(47)	14(20)		4(15)	1(3)	8(23)	3(18)	
Fetal heart rate (bpm)										
< 130	0(0)	2(12.5)	4(25)	1(4)	0.729	5(28)	3(20)	1(13)	2(22)	0.013
130–140	0(0)	12(75)	11(69)	19(83)		12(67)	6(40)	7(87)	6(67)	
≥ 140	0(0)	2(12.5)	1(6)	3(13)		1(5)	6(40)	0(0)	1(11)	

*Note*: *ml* milliliters, *bpm* beats per minute

The adjusted multiple regression models are presented in Table 3. Compared to mothers under 18, infant deaths was less likely to occur in mothers in all the other age categories. Infants born to mothers with parity-four or more during NHIS were twice more likely to die compared to infants born to mothers with no parity during both cash and carry and NHIS. In both the cash and carry and NHIS periods, infants born vaginally were significantly less likely to die than infants born by Caesarean method.

**Table 3:** Adjusted multivariate logistic regression of perinatal mortality in infants born during the Cash & Carry and NHIS systems in Northern Region

Variables	Cash & Carry period	NHIS period
	Adjusted†	Adjusted†
	OR 95% CI	OR 95% CI
Maternal age		
< 18 (RC)	1	1
18–24	0.41 [0.18, 0.88]*	0.62 [0.18, 2.07]
25–34	0.44 [0.20, 0.96]*	0.56 [0.17, 1.85]
35 >	0.72 [0.33, 1.61]	0.69 [0.20, 2.35]
Maternal parity		
None (RC)	1	1
One	0.73 [0.50, 1.06]	1.10 [0.70, 1.70]
Two	0.77 [0.51, 1.16]	1.24 [0.76, 2.02]
Three	1.87 [1.33, 2.63]***	1.51 [0.88, 2.60]
Four-plus	1.61 [1.18, 2.21]**	2.12 [1.23, 3.64]**
Type of birth		
Caesarean (RC)	1	1
Vagina	0.41 [0.32, 0.54]***	0.60 [0.44, 0.83]**
Nature of delivery		
Twins (RC)	1	1
Singleton	0.41 [0..30, 0.57]***	1.34 [0.77, 2.34]
Sex		
Male (RC)	1	1
Female	1.08 [0.85, 1.35]	0.96 [0.72, 1.28]
Blood loss after delivery		
< 200 (RC)	1	1
200–250	0.61 [0.43, 0.85]**	1.04 [0.69, 1.57]
> 250	1.81 [0.57, 1.16]	1.62 [0.89, 2.93]
Fetal heart rate (bpm)		
< 130 (RC)	1	1
130–140	0.80 [0.35, 1.80]	0.40 [0.20, 0.80]*
> 140	0.29 [0.29, 1.67]	0.85 [0.33, 2.14]

*Note. OR* odds ratio, *CI* confidence interval, *RC* reference category; *p < 0.05; **p < 0.01; ***p < 0.001, *p*-values obtained from multivariable binary logistic regression analysis (Wald test statistic); † = Adjusted for all other variables in the model known to influence the outcome

## Discussion

This cross-sectional study compared trends in perinatal mortality among infants born during delivery fee exemption under the NHIS and infants born during health user fee paying period (Cash & Carry) at a tertiary health facility in the Northern Region of Ghana. The overall study results showed that on average, perinatal mortality had declined by almost half from the Cash and Carry period in the early 2000 through 2003 to the NHIS period in the years 2008 through 2011 when delivery fee exemption program was fully in place.

Except mothers 35 years or older, there were no significant differences observed in perinatal deaths in all the age categories. Mothers under 18 years, who are generally prone to poor birth outcomes, including infant deaths, were observed in this study to have had little perinatal deaths, especially in mothers who delivered during the NHIS. First time mothers (parity – none) experienced more perinatal deaths in both periods compared to mothers with one or more prior birth experiences. This finding was similar to findings in other studies done among adolescent mothers in sub-Saharan Africa and in Bangladesh [23, 24]. That first birth carries a higher risk of neonatal mortality has been attributed to inexperience in childbearing [24]. However, in the adjusted multiple regression models, infants born to mothers with four or more prior birth experience had lower odds of survival compared to infants born to first time mothers. The findings in the adjusted regression model may be indicative of some risks associated with multiparous women in Northern Region, especially since the odds for infant survival decreased further even after the women had the opportunity to deliver at a health facility during a fee-free delivery period.

During the Cash and Carry era, Caesarean births as a delivery option were in limited use compared to the NHIS era. Caesarean births in the Cash and Carry period were just below the WHO recommended upper-limit of 15% of all births. However, during the NHIS era, Caesarean births were increasingly being used as a birth option, which far exceeded the WHO recommended upper-limit stated earlier. This finding was consistent with findings from others studies that showed increased use of Caesarean births during the NHIS period in Ghana and the U.S. between 2003 and 2009, the study period [25, 26]. During the NHIS era when delivery fee was exempted, the number of Caesarean births and the proportion of perinatal deaths had both increased. Although perinatal deaths among the Caesarean births increased in both the “Cash and Carry” system (16%), and the NHIS period (24%), the rate of increase was significant in the latter. This observation was consistent with another study that found an increase in the Caesarean birth rates in Ghana [27]. The increase in both the Caesarean births rate and perinatal death rates during the NHIS period compared to the Cash and Carry could be due to the fact that complicated cases which hitherto could not have accessed health care because of financial barriers are now able to do so. Anecdotal information also indicates that the inception of the NHIS has led to pregnant women who previously patronised private facilities where they paid out of pocket to shift to public facilities. This has led to long waiting periods for caesarean section thereby increasing the decision to operation time. These delays could lead to perinatal deaths for compromised fetuses.

Mothers with a post-delivery blood loss of more than 250 ml were observed to have had higher perinatal mortality rates during the Cash and Carry system compared to similar mothers in the NHIS system. This finding is consistent with observations made by the World Health Organization in Burkina Faso, another sub-Saharan African country [28].

### Limitations

Although the overall study findings demonstrate that perinatal mortality during the NHIS period had declined, the study has several limitations. The overall reduction in the mortality rate over time could not have been entirely influenced by the availability of the NHIS as the main factor responsible for this reduction. Rather, there is the possibility that other factors such as: health facility improvement to upgrade the Northern Region hospital to a teaching hospital beginning in 2005; increased staff strength to the status of a teaching hospital; and improved quality of intrapartum and neonatal care, which could all have contributed to the overall reduction in the perinatal mortality in the region. Other limitation of the study included availability of fewer birth records for the year 2000 in relation to the remaining other years (2001–2003) during the Cash and Carry period. Additionally, the lack of information on fetal gestational age was a major limitation in this study. Gestational age as a major explanatory factor in infant deaths has already been established in other studies [29, 30]. However, we do not have any reason to believe that the gestational ages in the two eras differed. Furthermore, the lack of complete pregnancy history such as, maternal weight gain and morbidity during pregnancy that could have helped explored any linkage was a major limitation in our study. Another limitation includes the lack of information on the marital status, employment, income or education levels of the mothers. The link between socioeconomic status and delivery outcomes, which has already been established in the literature, would have explained the differences in perinatal deaths and the selected variables observed in this study during the cash and carry and NHIS periods [31, 32]. Since only hospital birth records were purposively sampled for the study, there is a chance that our results may have been biased because records extracted from either the Cash and Carry or the NHIS eras may have included that of mothers with high risk pregnancies that had potential for negative birth outcomes. We therefore cannot conclusively indicate that the high risk pregnancies were higher in one group or the other. Additionally, despite the availability of the NHIS, some women in the entire Northern Ghana still have cultural preference for home births, hence our sample, and for that matter our results did not include perinatal mortalities in the population that chose home births even if they accessed NHIS for antenatal care services.

Although the hospital serves residents of Ghana’s poorest region, which enabled us to assume that mothers who gave birth in the region had similar socioeconomic status, data on specific differences in the SES of the mothers who gave birth at the hospital might have yielded additional information regarding any observed differences in infant deaths.

In spite of the numerous limitations in this study, it is important to acknowledge that the availability of the delivery exemption program through the NHIS provided poor women in the region access to antenatal care and skilled professional services before and during delivery of their babies, which contributed to the reduction in overall perinatal deaths observed in this study, from 8% during the Cash and Carry era to 4% in the NHIS era.

## Conclusion

As observed in this study, overall perinatal mortality rates among infants born during the Cash and Carry era in 2000–2003 declined by 50% when the NHIS delivery fee exemption program was in effect in 2008–2011. Perinatal mortality was observed to have declined in mothers age 35 or older, those with three or more prior birth experiences and mothers with a postpartum blood loss of 250 ml or higher during the NHIS period compared to Cash and Carry era at this tertiary hospital. Caesarean births increased during the NHIS era compared to the Cash and Carry era. These findings suggest that pregnant women in the Northern Region of Ghana may have utilized the opportunity to access services through the NHIS exemption program for skilled care at a tertiary hospital for delivery of their infants. The use of skilled care at delivery in Ghana is largely known to be significantly lower compared to use of antenatal care services in the current NHIS system. Therefore, the findings from this study suggests that implementation of the NHIS delivery fee exemption program may have contributed to the country’s progress towards meeting the MDG 4, (i.e. reducing child under-five deaths by two-thirds). In fact, findings from this study should guide maternal and child health policy makers and frontline health workers to ensure that vulnerable groups with poor birth outcomes utilize the NHIS delivery fee exemption services for improved birth outcomes.

## Abbreviations

LMIC: Low -middle income countries
MDG: Millennium Development Goals
MPI: Multi-dimensional Poverty Index
NHIS: National Health Insurance Scheme
TTH: Tamale Teaching Hospital
WHO: World Health Organization
